# Supplementary material for: Protocol of a prospective comprehensive evaluation of an elastic band beard cover for filtering facepiece respirators in healthcare
Source: PLoS One. 2023 Jan 31;18(1):e0281152. doi: 10.1371/journal.pone.0281152 (PMC9888701; doi:10.1371/journal.pone.0281152)
Supplement: S1 File — Baseline survey. (PDF) [file pone.0281152.s001.pdf]

Demographic Details

By completing this survey, you are consenting to quantitative fit testing of N95 respirators, and you understand that your de-identified information may be used for quality assurance and HREC-approved research purposes.

Thank you

Basic Demographics

Timestamp

____

Full name (first name and surname)

____

Health Service/Employer

____

RMH Employee Number (if applicable)

____

Date of birth

____

Gender

○ Male ○ Female ○ Other

Height (cm)

____

Weight (kg)

____

Role

____

Area/Department

____

Years of healthcare experience

____

Mobile Number

____
(This will be used to send research surveys & appointment details)

Work Hazard Assessment

When working in respiratory protective equipment (RPE) in your area, is there any temperature exposure > 30 degrees Celsius for periods greater than 15 minutes?

◯ Yes ◯ No

When working in your RPE, do your clinical tasks involve any very high-intensity activity (>6 metabolic equivalents [METS]),

☐ Heavy cleaning for greater than 30 minutes at a time
☐ Lifting heavy loads (>20kg for greater than 2 minutes) several times per shift
☐ Fast-paced walking for greater than 10 minutes duration, several times per shift
☐ None of the above

Are you involved in aerosol-generating (AG) procedures or in contact with patients with AG behaviours?

◯ Yes ◯ No

Medical/Health Safety Screen

Do you have significant claustrophobia that could limit your ability to wear a face mask?

◯ Yes ◯ No

Do you have lung, heart or other problems that cause limitation in ordinary physical activity, (e.g. shortness of breath when walking, or climbing stairs) or that could impair your ability to perform your usual roles at work?

◯ Yes ◯ No

Do you have any dizziness, shortness of breath, pounding in your chest, chest pain or other work-limiting symptoms when performing your usual tasks at work?

◯ Yes ◯ No

Do you have any neck or upper limb issue that would impair your ability to fit or wear a face mask at work?

◯ Yes ◯ No

Do you have to wear corrective lenses at work?

◯ Yes ◯ No

Do you wear

◯ Glasses ◯ Contact Lenses

Training & Experience with N95/P2 Masks

Have you been provided with formal education & training on respiratory protective equipment in the past 12 months?

◯ Yes ◯ No

How long ago? (months) 1-12 ____________________

In what format was the education delivered?
- ☐ Information brochure with instructions for use
- ☐ Face to face learning
- ☐ Online education
- ☐ Other

Please specify ____________________

Did you receive any performance assessment on your ability to safely don/doff and complete a user seal (fit) check of N95 masks?

◯ Yes ◯ No

Who performed this assessment?
- ☐ Infection Prevention (IPSS)
- ☐ Area/Department
- ☐ Clinical Education (medical, nursing, allied health)
- ☐ Other

Over the past 3-6 months, have you had to undertake alternate duties due to a lack of suitable respiratory protective equipment for bearded workers?

◯ All of the time ◯ Most of the time ◯ Some of the time ◯ Rarely ◯ Never

Over the past 3-6 months have you been wearing respiratory protective equipment (e.g. N95 mask) over your beard to protect yourself from SARS CoV-2?

◯ All of the time ◯ Most of the time ◯ Some of the time ◯ Rarely ◯ Never

How many N95 masks did you use whilst working in your current role?

in the last 4 weeks
- ◯ 0
- ◯ 1-10
- ◯ 11-20
- ◯ 21-30
- ◯ >30

in the last 3 months
- ◯ 0
- ◯ 1-20
- ◯ 21-40
- ◯ 41-60
- ◯ >60

What is your preferred model?	◯ Semi-rigid Cup: 3M 1860<br>◯ 3-panel flat-fold: 3M Aura 1870+<br>◯ 3-panel flat-fold: Trident<br>◯ Duckbill: Halyard Fluidshield<br>◯ Duckbill: BSN Proshield<br>◯ Flat Fold Cup: Care Essentials MSK-002<br>◯ Flat Fold Cup: BYD DE2322<br>◯ Other
Please specify	__________________________________
What size?	◯ Small ◯ Regular/ Medium

Personal/subjective opinions

	Strongly disagree	Disagree	Neutral	Agree	Strongly agree
I am concerned that I may contract COVID-19.	○	○	○	○	○
If I had concerns about respiratory protective equipment, I would feel confident to speak up and also that my concerns would be taken seriously by my direct supervisor/manager.	○	○	○	○	○
I believe that my health service/employer has a strong safety culture with respect to respiratory protection and respiratory protective equipment	○	○	○	○	○
I believe I have adequate knowledge about respiratory protective equipment.	○	○	○	○	○
I feel I have received adequate training on proper use of respiratory protective equipment.	○	○	○	○	○
I feel confident with safe donning of N95 face filtering respirators/masks.	○	○	○	○	○
I feel confident with safe doffing of N95 face filtering respirators/masks.	○	○	○	○	○
I feel confident performing a user seal (fit) check each time I wear a N95 face filtering respirators/mask.	○	○	○	○	○
I believe I should have regular quantitative fit testing.	○	○	○	○	○
I feel that the N95 respirator I wear over my beard provides sufficient protection from respiratory hazards.	○	○	○	○	○
