# Supplementary material for: Protocol of a prospective comprehensive evaluation of an elastic band beard cover for filtering facepiece respirators in healthcare
Source: PLoS One. 2023 Jan 31;18(1):e0281152. doi: 10.1371/journal.pone.0281152 (PMC9888701; doi:10.1371/journal.pone.0281152)
Supplement: S2 File — Principles of Standard of Operation. (PDF) [file pone.0281152.s002.pdf]

<u>Principles to develop standard of procedure for elastic band beard cover technique:</u>

1. **Choice of elastic band:**

Predefined criteria for physical properties:

- Elasticity and recoil: repeatable and reliable elongation force
- Preferably non-latex
- Non-permeable

Manufacturer:

- Must be produced by a reputable manufacturer with an established product currently used in healthcare.

Single use:

- Single use, due to lack of evidence that can prove materials do not deteriorate over time and are able to withstand multiple wash and disinfection cycles.

Minimise modification of product:

- Longitudinal cutting is not permitted, as it may reduce integrity of the product, as well as result in a non-standardised sized elastic band.

2. **Choice of filtering facepiece respirator (FFR):**

- Should only be used with high quality FFRs, which have high quantitative fit test pass rates, high user assessments, and are readily available.

3. **Principles for a safe repeatable technique:**

- Width of any head covering (e.g. Turban) must be minimised to reduce the likelihood of a gap between face and elastic band.
- Minimise total head circumference including all head coverings, so that circumferential head straps of FFRs are not overstretched.

- A disposable head cover needs to be worn to cover the knot of the elastic band, to minimise the risk of entanglement with the head straps of FFR and to simplify the doffing technique (please refer to the next point).
- Doffing must be simple, as it usually occurs at a time of high cognitive load. The technique is designed to be performed without the assistance of a second person (therefore no knots to untie).
- Must meet Infection Prevention and Surveillance standards and have local governance approval.
- The technique must be standardised to minimise variation within and between users, so that respiratory protection can be assured and that trained personal protective equipment (PPE) spotters can accurately assess safety.
- User must demonstrate skill competency.
- User must be able to repeatedly pass quantitative fit tests before clinical deployment.
- User must regularly be re-assessed to ensure ongoing safety and provide user assessments to ensure that the elastic band beard cover does not interfere with occupational activities, and is comfortable and tolerable for the duration of wear.
