# Supplementary material for: Protocol of a prospective comprehensive evaluation of an elastic band beard cover for filtering facepiece respirators in healthcare
Source: PLoS One. 2023 Jan 31;18(1):e0281152. doi: 10.1371/journal.pone.0281152 (PMC9888701; doi:10.1371/journal.pone.0281152)
Supplement: S3 File — Skill assessment marking sheet. (PDF) [file pone.0281152.s003.pdf]

Skill Assessment

Record ID	__________________
Date of the test	__________________
Name	__________________
Health service/Employer	__________________
Employee number	__________________
Fit tester	__________________
Mask used in skill assessment	◯ 3M 1870+ Aura<br>◯ Industree Trident

Preparation

Mark scores with either 0 or 1 where 0 = incorrect and 1 = correct.

Prepare head covering by making it as small a profile as possible. If possible, remove the outer turban (Pagri) leaving an under-turban headcloth/cover (Patka)	◯ 0 ◯ 1
Prepare beard by making it as smooth and regular as possible, including use of hair tie on the beard (high up on the neck) for beard lengths over 8 cm	◯ 0 ◯ 1
Perform hand hygiene	◯ 0 ◯ 1
Visual inspection of Theraband cut to 1m length	◯ 0 ◯ 1
Visual inspection of respirator	◯ 0 ◯ 1
Ensure a mirror is available for donning	◯ 0 ◯ 1
Total Score	__________________<br>(/6)

Donning the Theraband and a 3-panel FFR

Place Theraband under the chin and stretch up over the head approximately 3cm anterior to the crown and tie in a double knot	◯ 0 ◯ 1
Ensure the tightness & stretch is adequate so the Theraband is not gaping	◯ 0 ◯ 1
Ensure Theraband creates a smooth artificial skin with no creases and minimal facial hair in the face seal section of the respirator	◯ 0 ◯ 1
Place a hair cover over the Theraband	◯ 0 ◯ 1
Fully open the top and bottom panels, bending the nosepiece to create a gentle curve. Make certain the bottom panel is unfolded and completely opened	◯ 0 ◯ 1
Place the respirator on your face so that the bottom panel is securely under the Theraband & chin and so that the foam rests on the nasal bridge, covering the nose	◯ 0 ◯ 1
Position the upper strap at the crown of the head, so that it runs above the ears	◯ 0 ◯ 1
Position the bottom strap around the neck and below the ears	◯ 0 ◯ 1
Adjust for a comfortable fit by pulling the top panel toward the bridge of the nose and the bottom panel under the chin to maximise vertical height	◯ 0 ◯ 1
Make sure that the edges of the respirator are not curled under and that the mask is symmetrical on the face	◯ 0 ◯ 1
Place the fingertips from both hands at the top of the metal nose bar, then slide the fingertips down both sides of the metal strip to mould to the shape of the nose	◯ 0 ◯ 1
Total Score	______________________ (/11)

User Seal Check

Place hand(s) over the respirator front panel so that airflow is limited to the periphery (face-seal section) of the mask, taking care to not push the mask onto the face and to avoid moving the respirator on the face	◯ 0 ◯ 1
Negative pressure seal: Take a quick breath in to check whether the respirator seals tightly to the face by either drawing in slightly to the face (collapsible respirators e.g. duckbill) or the absence of an air leak around the face seal (non-collapsible e.g. Aura or Trident)	◯ 0 ◯ 1
Positive pressure seal: gently exhale. If you feel an obvious leak there is not a proper seal	◯ 0 ◯ 1
Total Score	__________________ (/3)

Doffing the Theraband and a 3-panel FFR

Perform hand hygiene	◯ 0 ◯ 1
Remove (or reposition) the bottom strap by pulling over the back of your head (to either join the top strap or to drop in front of your mask)	◯ 0 ◯ 1
Grab the top strap(s) being careful not to touch your face or mask	◯ 0 ◯ 1
Lean slightly forward, moving the strap over your head and allow the respirator to fall away from your face	◯ 0 ◯ 1
Only touch the straps of the respirator, do not touch any part of the filter panels	◯ 0 ◯ 1
Discard the respirator into a clinical waste bin	◯ 0 ◯ 1
Perform hand hygiene	◯ 0 ◯ 1
Remove the head cover (if not already doffed)	◯ 0 ◯ 1
Perform hand hygiene	◯ 0 ◯ 1
Grab the top of the Theraband straps on either side of the crown of the head (without undoing the knot)	◯ 0 ◯ 1
Lean slightly forward, moving the strap over your head and allow the Theraband to fall away from your face	◯ 0 ◯ 1

Discard in a clinical waste bin	◯ 0 ◯ 1
Perform hand hygiene	◯ 0 ◯ 1
Total Score	______________________ (/13)
