# Supplementary material for: Protocol of a prospective comprehensive evaluation of an elastic band beard cover for filtering facepiece respirators in healthcare
Source: PLoS One. 2023 Jan 31;18(1):e0281152. doi: 10.1371/journal.pone.0281152 (PMC9888701; doi:10.1371/journal.pone.0281152)
Supplement: S4 File — Usability assessment survey. (PDF) [file pone.0281152.s004.pdf]

Usability Assessment

We would like to know your experience with the TheraBand and N95 respirator after your fit testing. Please answer the following questions.

Timestamp	__________________________
Name	__________________________
Name of health service/Employer	__________________________
Date of Birth	__________________________
How many times have you used a TheraBand in a clinical setting?	◯ Never (only in training)<br>◯ 1-5<br>◯ 6-10<br>◯ 11-20<br>◯ 21-40<br>◯ >40
What is the usual duration of wear in hours before doffing each time?	◯ Not applicable (only used in training)<br>◯ < 1<br>◯ 1-2<br>◯ >2-4<br>◯ >4-6<br>◯ >6
What is your preferred model of respirator to wear with a TheraBand?	◯ 3-panel flat-fold: 3M Aura 1870+<br>◯ 3-panel flat-fold: Trident

	Strongly disagree	Disagree	Neutral	Agree	Strongly agree
I feel that the education and training was adequate regarding the use of the TheraBand.	◯	◯	◯	◯	◯
I am comfortable with the preparation and inspection of my TheraBand.	◯	◯	◯	◯	◯
I believe I can repeatedly safely don my TheraBand and respirator.	◯	◯	◯	◯	◯
I find the TheraBand and respirator provides a good seal.	◯	◯	◯	◯	◯
I do NOT get too hot when wearing my TheraBand and respirator.	◯	◯	◯	◯	◯

I do NOT perspire (sweat) excessively when wearing my TheraBand and respirator.	○	○	○	○	○
I am able to hear adequately while using my TheraBand and respirator.	○	○	○	○	○
I am able to speak clearly and be understood while using my TheraBand and respirator.	○	○	○	○	○
The TheraBand is not interfering with the rest of my personal protective equipment (PPE).	○	○	○	○	○
I believe I can safely doff my TheraBand.	○	○	○	○	○
I feel well protected from respiratory hazards while wearing the TheraBand and respirator.	○	○	○	○	○

Do you think you pass the user-seal (fit) check when wearing your TheraBand under your respirator?

- ○ Every single time
- ○ Most of the time
- ○ Sometimes
- ○ Rarely
- ○ Never

How would you describe the firmness of the fit of the TheraBand on the face?

- ○ Too tight
- ○ About right
- ○ Too loose

How would you describe the breathability of your respirator when using the TheraBand?

- ○ Poor
- ○ Average
- ○ Good

Have you ever had any of the following problems whilst using your TheraBand and respirator?

Eye or periorbital irritation limiting the duration of use	○ Yes ○ No
Skin irritation or rash limiting the duration of use or requiring special skincare	○ Yes ○ No
Anxiety limiting the duration of wear	○ Yes ○ No

Significant pressure areas that have limited your duration of use of the mask

Nose	○ Yes ○ No
Cheeks	○ Yes ○ No
Ears	○ Yes ○ No

Chin	◯ Yes ◯ No
Other - Please specify	__________________________

	Very poor	Poor	Average	Good	Very good
How would you rate the overall comfort (feel) and tolerability of the TheraBand and respirator combination?	◯	◯	◯	◯	◯
Overall how would you rate the overall ease of use of the TheraBand and respirator combination?	◯	◯	◯	◯	◯
What is your overall assessment of the combination of TheraBand and your preferred respirator?	◯	◯	◯	◯	◯

List any work tasks that you are unable to do using the TheraBand and respirator combination:

Using a stethoscope?	◯ Yes<br>◯ No<br>◯ NA
Wearing spectacles/glasses	◯ Yes<br>◯ No<br>◯ NA
Other tasks - please provide details	__________________________
Please feel free to provide any other comments:	__________________________
