# Supplementary material for: Protocol of a prospective comprehensive evaluation of an elastic band beard cover for filtering facepiece respirators in healthcare
Source: PLoS One. 2023 Jan 31;18(1):e0281152. doi: 10.1371/journal.pone.0281152 (PMC9888701; doi:10.1371/journal.pone.0281152)
Supplement: S5 File — (PDF) [file pone.0281152.s005.pdf]

QA2022022 QA Acknowledgement

Forza (Bragato), Angela

Wed 3/16/2022 3:06 PM

To:Williams, Daryl <Daryl.Williams@mh.org.au>; Ng, Irene <Irene.Ng@mh.org.au>;

Cc:Begg, Fiona <fiona.begg@mh.org.au>; Bodas, Charles <Charles.Bodas@mh.org.au>; Kave, Benjamin <Ben.Kave@mh.org.au>;

8 attachments

QA Application Form_Elastic Band Singth Thattha_04022022.doc; QA protocol_Theraband_Feb2022.docx; Appendix 3_Theraband respirator usability assessment_08.02.22.docx; Appendix 4_Theraband Research - HCW unable to shave - information sheet_8.2.2022.docx; Appendix 5_Theraband technique and QNFT procedure_Feb2022.docx; Appendix 1_Screening survey questions_FFRs Theraband_08.02.22.docx; Appendix 2_Skill assessment_Theraband & Respirator 08.02.22.docx; QA HOD Approval and Declaration Form_Feb2022.docx;

Dear Prof Williams,

QA2022022 titled: Respiratory Protection Program evaluation of the under-respirator elastic band beard cover (Singh Thattha technique) at Royal Melbourne Hospital

The project has been reviewed against the tenets of the National Statement on Ethical Conduct in Research 2007 (updated 2018) and the NHMRC Ethical Considerations in Quality Assurance and Evaluation Activities (March 2014).

This project has been assessed by a member of the Office for Research Ethics & Governance team and a member of the Royal Melbourne Hospital (RMH) HREC, as evaluation activity not requiring Human Research Ethics Committee (HREC) review.

Site - Royal Melbourne Health VIC

Documents noted:

- QA Application Form signed by Project Lead 04/02/2022
- Protocol Version 1 dated 01/02/2022
  - Appendix 1 - Screening survey questions_FFRs Theraband_08.02.22
  - Appendix 2 - Skill assessment_Theraband & Respirator 08.02.22
  - Appendix 3 - Theraband respirator usability assessment_08.02.22
  - Appendix 4 - Theraband Research - HCW unable to shave - information sheet_8.2.2022
  - Appendix 5 - Theraband technique and QNFT procedure_Feb2022
- QA Head of Department Approval and Declaration Form signed 04.02.2022

Other information about your QA approval:

Your project number is **QA2022022.** Please quote this number in future correspondence

- If there are any changes to the project a new QA project will need to be submitted
- If the study is to continue beyond 2 years, a new QA project will need to be submitted
- Annual progress reports are not required for QA projects.

- All documentation regarding this project, including this email, must be kept for 12 months from completion.
- However if you intend to publish the results, documentation must be kept for 5 years post publication or 5 years from the decision not to publish.

Note for multisite projects:

- This assessment is for RMH sites only. For other sites, the organisation may accept this assessment or choose to undertake another assessment.
- Site Governance Authorisation is required at each site participating in the study before the research project can commence at that site.

PLEASE FILE THIS EMAIL WITH THE PROJECT RECORDS.

Regards,

The Royal Melbourne Hospital

Angela Forza - Manager Human Research Ethics Committee
Office for Research, RMH City Campus, Level 2 South West
300 Grattan Street I Parkville Victoria 3050
angela.forza@mh.org.au I **thermh.org.au** I (P) 9342 8530

The Royal Melbourne Hospital acknowledges the Kulin nations as the Traditional Custodians of the land on which our services are located.

We are committed to improving the health and wellbeing of Aboriginal and Torres Strait Islander peoples.

WORKING DAYS: Part Time - Tues & Wed

New Process: Streamlined Annual Reporting

Single due date for all projects

Effective immediately annual reports for **ALL** projects (Reviewing HREC and Site Specific) are now due **31 March each year**.

For full details including what this change means for you project refer to the OFR website.
