# Supplementary material for: Protocol of a prospective comprehensive evaluation of an elastic band beard cover for filtering facepiece respirators in healthcare
Source: PLoS One. 2023 Jan 31;18(1):e0281152. doi: 10.1371/journal.pone.0281152 (PMC9888701; doi:10.1371/journal.pone.0281152)
Supplement: Attachment — Submitted filename: Response to Reviewers.docx [file pone.0281152.s006.docx]

**Response to Reviewers**

**Journal Requirements:**

**When submitting your revision, we need you to address these additional requirements.**

**1. Please ensure that your manuscript meets PLOS ONE's style requirements, including those for file naming. The PLOS ONE style templates can be found at 
https://journals.plos.org/plosone/s/file?id=wjVg/PLOSOne_formatting_sample_main_body.pdf and 
https://journals.plos.org/plosone/s/file?id=ba62/PLOSOne_formatting_sample_title_authors_affiliations.pdf**

***Response:*** Apologies. We have now revised the manuscript so that it meets PLOS ONE’s style requirements.

**2. Your ethics statement should only appear in the Methods section of your manuscript. If your ethics statement is written in any section besides the Methods, please move it to the Methods section and delete it from any other section. Please ensure that your ethics statement is included in your manuscript, as the ethics statement entered into the online submission form will not be published alongside your manuscript.**

***Response:*** We have moved the ethics statement to the Methods section. Thank you for the suggestion.

**3. We note that Figure 1 includes an image of a participant in the study. 
As per the PLOS ONE policy (http://journals.plos.org/plosone/s/submission-guidelines#loc-human-subjects-research) on papers that include identifying, or potentially identifying, information, the individual(s) or parent(s)/guardian(s) must be informed of the terms of the PLOS open-access (CC-BY) license and provide specific permission for publication of these details under the terms of this license. Please download the Consent Form for Publication in a PLOS Journal (http://journals.plos.org/plosone/s/file?id=8ce6/plos-consent-form-english.pdf). The signed consent form should not be submitted with the manuscript, but should be securely filed in the individual's case notes. Please amend the methods section and ethics statement of the manuscript to explicitly state that the patient/participant has provided consent for publication: “The individual in this manuscript has given written informed consent (as outlined in PLOS consent form) to publish these case details”.**

**If you are unable to obtain consent from the subject of the photograph, you will need to remove the figure and any other textual identifying information or case descriptions for this individual.**

***Response:*** We have obtained a signed consent from the individual whose image is shown in Figure 1. We have also included a statement to clarify this in the method section. **[Note: HTML markup is below. Please do not edit.]

Reviewers' comments:

Reviewer's Responses to Questions**

**Comments to the Author

1. Does the manuscript provide a valid rationale for the proposed study, with clearly identified and justified research questions?

The research question outlined is expected to address a valid academic problem or topic and contribute to the base of knowledge in the field.**

**Reviewer #1: Yes**

**Reviewer #2: Yes**

**Reviewer #3: Yes**

**2. Is the protocol technically sound and planned in a manner that will lead to a meaningful outcome and allow testing the stated hypotheses?

The manuscript should describe the methods in sufficient detail to prevent undisclosed flexibility in the experimental procedure or analysis pipeline, including sufficient outcome-neutral conditions (e.g. necessary controls, absence of floor or ceiling effects) to test the proposed hypotheses and a statistical power analysis where applicable. As there may be aspects of the methodology and analysis which can only be refined once the work is undertaken, authors should outline potential assumptions and explicitly describe what aspects of the proposed analyses, if any, are exploratory.**

**Reviewer #1: Yes**

**Reviewer #2: Yes**

**Reviewer #3: Partly**

**3. Is the methodology feasible and described in sufficient detail to allow the work to be replicable?


Descriptions of methods and materials in the protocol should be reported in sufficient detail for another researcher to reproduce all experiments and analyses. The protocol should describe the appropriate controls, sample size calculations, and replication needed to ensure that the data are robust and reproducible.**

**Reviewer #1: Yes**

**Reviewer #2: Yes**

**Reviewer #3: Yes**

**4. Have the authors described where all data underlying the findings will be made available when the study is complete?

The** [**PLOS Data policy**](https://secure-web.cisco.com/1shX4RiiMP2vQ626ivy1lHyDzT4MBU2uGGAnfUNyBSxw2iKSRCJFI7buzN3wI-HFCBbQXKOHrt5xDnTu49-f20OAleXXWAQOQRbHSHDwtBp6rjjDPy-NTwAiTcY0BOmvrmkX6fXNxM09dmTAbgV0WRxl7mprquiWJxxDtxqa8EOjYn2L_giMIzI3SEW0NV6bBjeepxIeHc9BIrXyipho6e2BOf-eVi9Hpeohr56zcEaPi5FYtOJmP7ShG-9iKt8GNh0bh5GL5iImixc4d8TUjvyxY8bZUjvGGZZVuEc4b0cTjs4EPkWjKDw3X50ZBlJVH/https%3A%2F%2Fjournals.plos.org%2Fplosone%2Fs%2Fmaterials-and-software-sharing) **requires authors to make all data underlying the findings described in their manuscript fully available without restriction, with rare exception, at the time of publication. The data should be provided as part of the manuscript or its supporting information, or deposited to a public repository. For example, in addition to summary statistics, the data points behind means, medians and variance measures should be available. If there are restrictions on publicly sharing data—e.g. participant privacy or use of data from a third party—those must be specified.**

**Reviewer #1: Yes**

**Reviewer #2: No**

**Reviewer #3: Yes**

**5. Is the manuscript presented in an intelligible fashion and written in standard English?

PLOS ONE does not copyedit accepted manuscripts, so the language in submitted articles must be clear, correct, and unambiguous. Any typographical or grammatical errors should be corrected at revision, so please note any specific errors here.**

**Reviewer #1: Yes**

**Reviewer #2: Yes**

**Reviewer #3: Yes**

**6. Review Comments to the Author

Please use the space provided to explain your answers to the questions above and, if applicable, provide comments about issues authors must address before this protocol can be accepted for publication. You may also include additional comments for the author, including concerns about research or publication ethics.

You may also provide optional suggestions and comments to authors that they might find helpful in planning their study.


(Please upload your review as an attachment if it exceeds 20,000 characters)**

**Reviewer #1: The authors have presented a protocol for a prospective multimodal evaluation of an elastic beard cover for filtering facepiece respirators (FFR) involving quantitative fit testing at 0,3 and 12 months. The protocol is well considered and presented clearly with appropriate justification, namely the lack of comprehensive evaluation of the suitability of elastic beard covers in healthcare settings. Relevant literature is cited.

I have only a few minor comments;
1. Methods p7. Is there methodological justification for the exclusion of healthcare workers who are not clean shaven for reasons other than religious, cultural or medical? This should be clarified.**

***Response:*** Use of tight-fitting respirators with facial hair is against international standards (OSHA Respiratory Protection Standard, 29 CFR 1910.134)^1^ and manufacturer’s instructions^2^. As shown in Prince et al.’s study^3^, facial hair under a P2/N95 respirator can diminish respiratory protection and potentially jeopardise healthcare workers’ and patient safety. We believe that requiring employees to shave for respiratory protection is a reasonable request under OHS law^4,5^. We should show compassion to healthcare workers who may experience a loss of identity or skin irritation with daily shaving. However, the use of elastic band beard cover technique should be prioritised for healthcare workers who cannot shave with tenable reasons, such as religious, cultural or medical restrictions, which are the inclusion criteria for this study. This helps ensure that this group of healthcare workers are not discriminated against and potentially alleviate pressure from workforce shortage.

We have added the justification for the exclusion criteria in the methods section of the protocol.

^1^ <https://www.cdc.gov/niosh/npptl/pdfs/facialhairwmask11282017-508.pdf>

^2^ <https://multimedia.3m.com/mws/media/1682579O/facial-hair-and-respirator-fit-testing-policy-technical-bulletin.pdf>

^3^ Prince SE, Chen H, Tong H, et al. Assessing the effect of beard hair lengths on face masks used as personal protective equipment during the COVID-19 pandemic. Journal of exposure science & environmental epidemiology. 2021:1-8.

^4^ <https://www.worksafe.vic.gov.au/occupational-health-and-safety-your-legal-duties>

^5^ https://www.legislation.vic.gov.au/in-force/acts/occupational-health-and-safety-act-2004/042

**2. Methods p6-. The authors may wish to consider grading or quantifying beard lengths unless they only intend to recruit participants with ‘full beards’, which should be explicitly stated if it is the case. Degree of facial hair has been correlated with FFR fit and may impact the study results.**

***Response:*** Thank you for the comment. Yes, we are measuring beard length each time the participant is having a quantitative fit test. Apologies for not mentioning this in the original protocol. We also believe that the density of the hair follicles and the grooming style may also have an impact on respirator fit. However, we are not collecting these data in this study. We anticipate that almost all our participants would be fully bearded because they would have grown their beard for a while with religious, cultural or medical restrictions.

We have now added beard length measurement as one of the data outcomes in the methods and statistical analysis sections of the protocol.

**Reviewer #2: Thank you for the opportunity to review this protocol. I found it well written, well-reasoned, and that the authors were forthcoming about any foreseeable limitations and how these are mitigated (e.g., the likely small sample sizes). The surveys were well constructed and, assuming sufficient sample sizes, I think they will address the stated study aims. I also think the focus of the proposed project is timely and important given the increased interest in bioaerosols and PPE efficacy that arose due to SARS-COV2.

My only minor critique has to do with the lack of information about data sharing. Data sharing/availability is key part of every PLOS review, and I found the statement “All relevant data from this study will be made available upon study completion” to be rather vague. Shared how? Via appendices? Via dryad? Will the data be shared publicly and freely without restriction? What does “relevant” mean? Given the choice to submit this protocol to PLOS, I think the authors need to add additional clarity on the scope and manner of their intended data sharing in the body of the protocol. Beyond this I have no further revisions to recommend.**

***Response:*** Thank you for the positive comments. Apologies for the vague data sharing statement. We have now revised the statement, which says - “All the individual de-identified data that support the findings of this proposed study will be available upon request from the corresponding author. Data will be available immediately following publication of the result findings until five years after publication. We have included this statement in the protocol.

**Reviewer #3: Although the study is technically sound, given the recent publication by Bhatia et al. (see Findings and citation below), my concern is that the research gap described on page 6 is no longer the primary motivation for the study. The abstract discussion also indicates “This is the first large-scale multimodal assessment in the literature of an elastic band beard cover for FFRs” which may no longer be accurate.
Given the potential for success with the elastic band technique and the models of respirator proposed to be tested (see data from table 2 below), the study might be characterized as a replication of an existing study, with a longer-term evaluation of efficacy.**

***Response:*** Thank you for the comments. We agree that our study proposal is no longer the first in the literature to assess quantitative fit test results of the elastic band beard cover technique with a reasonable sample size. Our study is different from Bhatia’s study in several ways. Firstly, unlike Bhatia’s study, our methodology is strictly standardized, including donning/doffing technique, training process, types of respirators used and sequence of the fit testing procedure. Moreover, we have other components in the evaluation, including skill assessment, usability assessment and repeated assessments over time. We anticipate that our sample size will be larger than Bhatia’s study as well. Therefore, we believe that the findings of our study will provide additional and valuable information in the literature.

We have now revised both the abstract and the main text discussion sections to ensure that we do not state our study is the first to perform fit test assessment of the elastic band beard cover technique.

**One idea for a novel intervention would be to test the use of a balaclava rather than an elastic exercise band (https://www.amazon.com/dp/B01LE02EHQ/). As someone who has worn the elastic band for testing and study purposes, user comfort is an issue to consider. The elastic band is not a garment intended to be worn and captures sweat with extended wear. It also can create discomfort based on positioning under the chin and how tightly it is knotted atop the head. On the other hand, a balaclava is intended to be worn for cold weather or adventure activities and may tolerate sweat and extended wear more favorably. It is a garment that can be worn in multiple styles (over the head or not, over the nose, under the chin) and so could be adjusted to cover the beard but not the nose and mouth of the user. Because it does not require tying or other modes of securing to the head, it may represent an easier to use intervention. Testing for whether this intervention works with disposable respirators and collecting information on the likelihood of usage, comfort, tolerability, etc. (either vs. an exercise band or alone) would represent a novel study proposal. Again, replication is important for science, but extending research and testing new ideas is also important.
*Response:*** Thank you for the suggestions. We agree that evaluating fit test results with the use of balaclava would be a novel intervention and that wearing a balaclava may be more comfortable than wearing an elastic band. We believe that elastic band is much more effective than balaclava because elastic band has an impermeable membrane, therefore acting like an artificial skin while providing an occlusive seal. In our protocol the elastic band is discarded after each use. On the other hand, a balaclava is permeable and can fold or create creases easily. Bhatia did measure the quantitative fit test pass rate with balaclava and found that the pass rate was only 17/30, 57%. (Ref: Appendix 4A in Clinical Excellence Commission. Respiratory Protection Program Manual. July 2022. Version 1.1. <https://www.cec.health.nsw.gov.au/__data/assets/pdf_file/0004/696712/Respiratory-Protection-Program-Manual.pdf>). We are hoping for QNFT pass rates to be greater than 80% using our standardised elastic band protocol and therefore, we think that the use of balaclava is probably not a technique that is broadly applicable for healthcare workers in clinical environment.

We have considered usability and safety while designing this study. We ensure that the participant will apply a disposable head cover over the elastic band before a respirator is donned, to cover the knot of elastic band so as to minimise the risk of entanglement with the head straps of the respirator, and also to minimise potential contamination of both the elastic band and the Turban or under-Turban (if present).

We acknowledge that elastic band may cause discomfort with extended use due to heat and moisture build up. We therefore included comfort and tolerability assessments in this study. Moreover, we are currently designing a separate study to measure heat and moisture levels with the use of elastic band under a respirator. We hope to gather more scientific information in this area.

**Findings: Thirty subjects were assessed; of these, 24 (80%) passed quantitative fit testing with at least one tight-fitting P2/N95 disposable respirator. Among these subjects, the median best-achieved fit factor was 200 (interquartile range 178-200). None of the subjects had an adverse reaction to the under-mask beard cover.
3M 1860 14/28 (50) 170 (128–200)
3M 1870+ 7/20 (35) 199 (181–200)
Trident RTCFFP2 15/22 (68) 200 (191–200)

Bhatia DDS, Bhatia KS, Saluja T, Saluja APS, Thind A, Bamra A, Singh G, Singh N, Clezy K, Dempsey K, Hudson B, Jain S. Under-mask beard covers achieve an adequate seal with tight-fitting disposable respirators using quantitative fit testing. J Hosp Infect. 2022 May 31;128:8-12. doi: 10.1016/j.jhin.2022.05.015. Epub ahead of print. PMID: 35662553.**

**7. PLOS authors have the option to publish the peer review history of their article (**[**what does this mean?**](https://secure-web.cisco.com/1aXg0GR4Fk9bcg7o5B-U8N3P1UrqDTAT5qr5q5hZPd6Td0ompY7t36uaF-pfzG2ZJy9ZQWW2p2kirFL23XOpNod3OB4mDP48mDz-B1IZG8DXSPgflp1R9FT7dgQVWoO6z5fsthCykwaGqLjGm4j05cJ2vxf-iAYOWHflMcjUkBYbFZUy7DUTDT4c7qZdKB1NzSSMgHVYG_1QTb6rsGIj7kVoWouoz6dAB9J7DwA8O8iO15rAZ-RRdzYN5IJVx0cAmp1OiBSg-QOSvgTDRJwzcOFXWCItKoGlWaE5WvIijxL2TRVtvyZRcm7J-49qHeHXX/https%3A%2F%2Fjournals.plos.org%2Fplosone%2Fs%2Feditorial-and-peer-review-process%23loc-peer-review-history)**). If published, this will include your full peer review and any attached files.


If you choose “no”, your identity will remain anonymous but your review may still be made public.


Do you want your identity to be public for this peer review? For information about this choice, including consent withdrawal, please see our** [**Privacy Policy**](https://secure-web.cisco.com/1DNleO27pS5L-AR7GoBlGlBiUfeuYHv7UkfiUUxH1SHlDHAi6lfBf2MsZv5PxSIzPerWSSAE0j5LjVNd4VVV1CrKOfaZzGIBfTs1eS81lgrOHCSlfUy9BW--nZXNWSjmCX4CkmHA7Dy4La7Uq4u_PJ5t0nxNpAiwRi0PkWQF3rTJngprMF_HePH7kSIM3Dz2PcYOQrqwhNLx1xfQrtu7Hn0QyuPjAHZ7GdzjiePTi3sMkbXC1lC5XVogrOJSKfTdPfunUpJkb2vfFhubAA3cMYDCTjL3lVXGwvQJkp5Px8K1JKkWmhjXdkZXgU_1t9w0k/https%3A%2F%2Fwww.plos.org%2Fprivacy-policy)**.**

**Reviewer #1: No**

**Reviewer #2: No**

**Reviewer #3: Yes: Steven E. Prince**
